# *Staphylococcus cohnii* Causing a Latent Endophthalmitis: A Case Report

**DOI:** 10.1155/crop/7433713

**Published:** 2025-05-20

**Authors:** Hassan Javed Ahmed, Christos Christakopoulos, Steffen Heegaard

**Affiliations:** ^1^Department of Ophthalmology, Rigshospitalet, Glostrup, Denmark; ^2^Department of Ophthalmology, Zealand University Hospital, Naestved, Denmark; ^3^Department of Pathology, Rigshospitalet, University of Copenhagen, Copenhagen, Denmark

## Abstract

**Objective:** We report a case of latent postoperative endophthalmitis caused by *Staphylococcus cohnii* in a 72-year-old woman.

**Observation:** The patient was referred to the department of ophthalmology with blurry vision in her right eye 12 days after phacoemulsification. The clinical examination showed signs of endophthalmitis. A vitreous tap, with intraocular injection of ceftazidime, was performed, and topical dexamethasone and tobramycin were initiated. The postoperative follow-up showed reduction in the intraocular reaction, and the patient was afterwards discharged. After 6 months, her private ophthalmologist referred the patient again with signs of uveitis and macular edema. The clinical examination showed hypopyon and infiltrates on the posterior lens capsule which led to a vitrectomy with the removal of the posterior lens capsule infiltrate. With no signs of improvement at the postoperative follow-up, the intraocular lens was removed.

**Results:** A pathological examination with H&E, Gram, and Periodic Acid Schiff showed gram-positive cocci in relation to the lens capsule. Polymerase chain reaction was performed, detecting DNA of *Staphylococcus cohnii*.

**Conclusion and Importance:** *Staphylococcus cohnii* should be considered in cases of latent endophthalmitis. *Staphylococcus cohnii* is a gram-positive coagulase-negative bacterium that produces biofilm. Biofilm can promote adherence to implants leading to failure of therapy.

## 1. Introduction

Endophthalmitis can occur as a sequela of intraocular procedures, trauma, or an endogenous source [1]. It is a rare but dreaded complication after intraocular surgery, with an estimated incidence rate of 0.05%–1.8% [1, 2], but with potentially devastating outcome. Early diagnosis and prompt treatment are crucial for outcome. Infection is the most common cause, with *Staphylococcus epidermidis* (70%) [3] being the most common pathogen. Here, we describe a case of latent endophthalmitis caused by *Staphylococcus cohnii*.

## 2. Case Presentation

**Figure 1:**
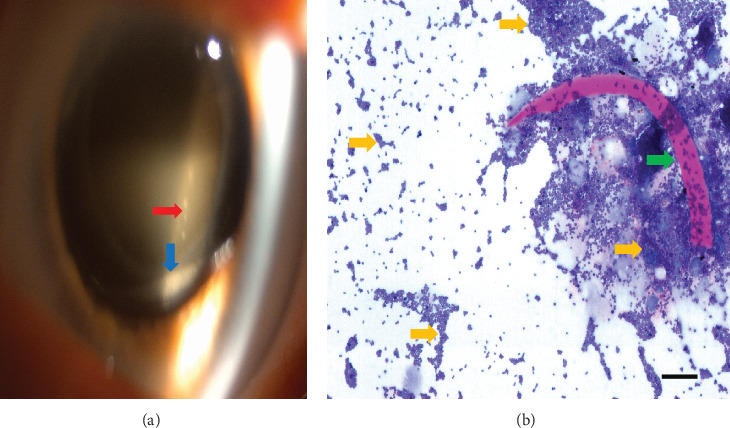
(a) Red arrow: fluffy infiltrates in posterior lens capsule. Blue arrow: hypopyon in the capsular bag. (b) PAS staining, bar = 250 *μ*. Several gram-positive cocci (*Staphylococcus cohnii*) (yellow arrow) are seen in close connection to the lens capsule (green arrow).

A 72-year-old woman was referred to our department with blurry vision on the right eye (RE) 12 days after phacoemulsification. Her best corrected visual acuity (BCVA) was 0.4 on the RE. The clinical examination showed ciliary injection, intraocular reaction (IOR) (10 cells/field, 1+), and vitritis without retinal hemorrhages. A vitreous tap was performed with intraocular injection of ceftazidime and vancomycin, and topical dexamethasone and tobramycin (Tobradex) were initiated. The postoperative follow-up showed reduction in the IOR in the anterior chamber and no vitritis. The microbiological analysis showed polymorphonuclear and mononuclear leucocytes. The patient was discharged with BCVA 0.5 on Tobradex. Six months later, she was referred again with uveitis and macular edema on the RE. She had been treated for 3 weeks with ketorolac (Acular) and dexamethasone (Maxidex) by her private ophthalmologist with no signs of improvement. BCVA on the RE was 0.5. Findings included IOR+2 and vitritis. Treatment with Acular and Maxidex was intensified to four times daily. After 6 weeks, fluorescein angiography was performed, suggesting inflammatory macular edema. The patient was treated with one peribulbar triamcinolone (40 mg) injection. Worsening of symptoms was recorded after 1 month, and biomicroscopy showed hypopyon in the capsular bag, fluffy posterior lens capsule infiltrates (Figure 1a), and dense IOR (> 20 cells/field 3+). Vitrectomy, with the removal of the posterior lens capsule infiltrate and intraocular injection of ceftazidime and vancomycin, was performed on suspicion of recurrence of endophthalmitis. Bacterial cultures returned negative, but pathologic examination and microscopy with H&E, Gram, and Periodic Acid Schiff staining showed gram-positive cocci in direct contact to the lens capsule (Figure 1b). Polymerase chain reaction (PCR) was then performed on the vitreous and lens capsule sample, detecting DNA of *Staphylococcus cohnii*. Subsequent intraocular injection with vancomycin was given 2 months later due to recurrence. Due to lack of improvement, the intraocular lens (IOL) and lens capsule were removed. Four months later, an anterior chamber IOL implant was inserted in the RE. Finally, after obtaining quiescence, the patient was discharged 3 months later with BCVA of 0.5 on RE.

## 3. Discussion

*Staphylococcus cohnii* is a gram-positive coagulase-negative member of the bacterial genus *Staphylococcus.* It is commonly found on human skin and mucous membranes [4]. It has rarely been described as an opportunistic pathogen, causing urinary tract infections, endocarditis, and bacteremia [5]. The increasing frequency of its detection as an opportunistic pathogen has been attributed to the bacteria's production of biofilm [6], thus promoting adherence to medical devices. Biofilm may protect bacteria against immunological host defence mechanisms and antimicrobial chemotherapy [7].

This case documents endophthalmitis due to *Staphylococcus cohnii*, which was confirmed with PCR and bacterial cultures of the IOL. Contrary to the typical course of acute endophthalmitis, this case did not manifest rapid exacerbation or permanent retinal damage. Bacterial culture of the microorganism was not possible on vitreous gel biopsy, probably because of biofilm coating. Microbiological diagnosis became possible with PCR examination of the vitreous gel samples, and cure was achieved only after IOL removal.

## 4. Conclusion

In conclusion, *Staphylococcus cohnii* is a rare causative agent in cases of latent endophthalmitis [8] and should be considered in cases not responding to intravitreal antibiotics. Hypopyon in the capsular bag is a rare manifestation of postoperative endophthalmitis [9], which in our case was assumed caused by biofilm producing bacteria. In such cases, removal of the implant should be considered.

## Data Availability

The data that supports the findings of this study are available from the corresponding author upon reasonable request.
